# Results from a World Health Organization pilot of the Basic Emergency Care Course in Sub Saharan Africa

**DOI:** 10.1371/journal.pone.0224257

**Published:** 2019-11-13

**Authors:** Andrea G. Tenner, Hendry R. Sawe, Stas Amato, Joseph Kalanzi, Muhumpu Kafwamfwa, Heike Geduld, Nikki Roddie, Teri A. Reynolds

**Affiliations:** 1 Emergency Department, University of California San Francisco, San Francisco, California, United States of America; 2 Emergency Medicine Department, Muhimbili University of Health and Allied Sciences, Dar es Salaam, Tanzania; 3 Department of General Surgery, University of Vermont, Newport, Vermont, United States of America; 4 Department of Emergency Medicine, Makerere University, Kampala, Uganda; 5 Cavendish University Zambia, Lusaka, Zambia; 6 Division of Emergency Medicine, University of Cape Town, Cape Town, South Africa; 7 Department for the Management of NCDs, Disability, Violence and Injury Prevention, World Health Organization (WHO), Geneva, Switzerland; Victoria University, AUSTRALIA

## Abstract

**Background:**

Frontline providers around the world deliver emergency care daily, often without prior dedicated training. In response to multiple country requests for open-access, basic emergency care training materials, the World Health Organization (WHO), in collaboration with the International Committee of the Red Cross (ICRC) and the International Federation for Emergency Medicine (IFEM), undertook development of a course for health care providers—*Basic Emergency Care*: *Approach to the acutely ill and injured* (BEC). As part of course development, pilots were performed in Uganda, the United Republic of Tanzania, and Zambia to evaluate course feasibility and appropriateness. Here we describe participant and facilitator feedback and pre- and post-course exam performance.

**Methods:**

A mixed methods research design incorporated pre- and post-course surveys as well as participant examination results to assess the feasibility and utility of the course, and knowledge transfer. Quantitative data were analyzed using Stata, and simple descriptive statistics were used to describe participant demographics. Survey data were coded and grouped by themes and analyzed using ATLAS.ti.

**Results:**

Post-course test scores showed significant improvement (p-value < 0.05) as compared to pre-course. Pre- and post-course questionnaires demonstrated significantly increased confidence in managing emergency conditions. Participant-reported course strengths included course appropriateness, structure, language level and delivery methods. Suggested changes included expanding the 4-day duration of the course.

**Conclusion:**

This pilot demonstrates that a low-fidelity, open-access course taught by local instructors can be successful in knowledge transfer. The BEC course was well-received and deemed context-relevant by pilot facilitators and participants in three East African countries. Further studies are needed to evaluate this course’s impact on clinical practice and patient outcomes.

## Background

Basic emergency care is essential at every level of a healthcare system [1]. Frontline providers around the world deliver emergency care every day, often without dedicated training in managing emergency conditions. The effectiveness of care delivered in limited-resource settings is often compromised by inadequate triage, failure to recognize and provide initial management for emergency conditions, and delayed referral to advanced care, all of which result in avoidable death and disability [1–4]. Emergency care training that is purpose-designed for low and middle income countries (LMICs) can improve provider skills, facilitate more efficient use of existing resources, and reduce morbidity and mortality [5–11] by ensuring timely access to life-saving treatments. However, access to comprehensive, high-quality and context-relevant emergency care educational materials is limited.

Existing emergency care courses are either limited in scope (focusing on only one aspect of emergency care) or access [12–22], costly, or otherwise unsuited for resource-constrained practice settings. Many courses are designed around clinical practices from high resource settings and are dependent on diagnostics or technology that are unavailable in most care settings globally [23]. Some proprietary emergency care training courses, such as BLS and ACLS, require certified instructors, linkage to existing training centres, and expensive materials, [24] effectively placing them out of reach for the majority of providers in LMICs. Finally, many existing courses fail to account for the reality that providers other than doctors deliver much of the emergency care around the world.

In response to requests from multiple countries and international partners for open-access, basic emergency care training materials, the World Health Organization (WHO), in collaboration with the International Committee of the Red Cross (ICRC) and the International Federation for Emergency Medicine (IFEM), undertook development of a course for frontline emergency care providers—*Basic Emergency Care*: *Approach to the acutely ill and injured* (BEC). The BEC course is designed for a range of providers who manage acute illness and injury with limited resources and teaches a systematic approach to the initial assessment and management of time-sensitive conditions where early intervention saves lives.

In late 2015, WHO partnered with the African Federation for Emergency Medicine to conduct pilot implementations of the BEC course in Uganda, United Republic of Tanzania, and Zambia to evaluate course feasibility and potential barriers to use. Assessments included pre- and post-course exams covering all content domains, skills assessments, workbook completion checks, and standardized assessment of capacity to manage case scenarios. Successful completion of all course components and a >75% mark on the post-test were required to pass the course. We report here on local facilitator and participant views on the appropriateness, acceptability, and utility of the course, as well as on participants’ pre- and post-course performance on content-based examinations.

## Methods

### Study design

This was a mixed methods analysis, incorporating facilitator and participant surveys and participant examination results before and after course implementation.

### Participants and study setting

Facilitators and participants were identified by local hospital administrators and local emergency care leaders, including those from the national Ministries of Health. Facilitators were selected based on leadership ability, prior teaching experience, and emergency care experience. Participants had no prior formal emergency care training, but all had caring for acutely ill and injured patients as their primary job. Trainers from AFEM, WHO and local university partners conducted a 2-day training of trainers on teaching methodology and delivery mechanisms for key BEC concepts, after which the newly-trained facilitators taught the course to local participants. Five BEC course pilots were executed in November 2015 to December 2015, including two in Dar es Salaam, United Republic of Tanzania; two in Kampala, Uganda; and one Lusaka, Zambia. Each BEC course was taught over four days, although the timing of the skills module was adjusted to accommodate logistical needs at each site (S1 Table). Courses were conducted in English and formally hosted by national emergency care professional societies and Ministry of Health agencies.

### BEC course

The WHO Basic Emergency Care Course as delivered in this pilot was a 4-day course comprised of didactic lectures, interactive workbook questions, case scenarios, and hands-on skills sessions. For this course, we delivered the didactics in 3 days and dedicated one day to skills practice. Skills were briefly demonstrated and the participants were then allowed to practice until they had mastered the skill, which was demonstrated practically by completing a set of critical actions as outlined in the manual. To complete the course, participants had to complete all activities in their workbooks, pass all skills tests through the performance of all critical actions, successfully outline the management of one case scenario, pass a post-test with a score of >75% and attend all sessions. The course is now available online in an open-source format [25].

### Data collection and processing

Pre- and post-course facilitator and participant surveys were administered to capture participants’ and facilitators’ perceptions of the quality, appropriateness, and utility of the course. Open-ended survey questions assessed perceptions of course strengths and weaknesses. Closed book multiple-choice examinations were administered to participants before and after the course. Facilitators submitted sheets summarizing participant workbook completion. Handwritten responses and exam scores were transcribed into text documents and spread sheets, respectively, and imported and analysed with Stata (version 14).

### Data analysis

Data from pre- and post-course examinations and quantitative survey data were analyzed in Stata, and simple descriptive statistics were used to describe participant demographics. A Wilcoxon signed-rank test was used to evaluate the difference between median pre- and post-test scores.

## Results

### Demographics of survey participants

A total of 17 facilitators and 59 participants were identified for five courses. Facilitators included nurses, general practitioners, and specialist emergency physicians. Participant cadres included clinical officers, assistant medical officers, medical officers, nurses and nursing assistants, employed in a range of health care settings, from health centre and rural district hospitals to tertiary referral hospitals (Table 1). The Tanzanian courses were conducted with a training of trainers for 8 facilitators who then taught 23 participants. Next, a training of trainers was held in Uganda and the 5 newly trained Ugandan facilitators were joined by two of the recently-trained Tanzanian facilitators to teach two courses for 24 participants. The last course was conducted in Zambia, and a training of trainers was conducted for 4 local facilitators who were then joined by two of the Ugandan facilitators to conduct a course for 12 participants. Table 2 describes participant and facilitator demographics. To avoid duplicate counting, the table lists facilitators only once by country of origin, though as described above, some newly trained facilitators assisted with courses in neighbouring countries.

**Table 1:** Participant and facilitator demographics.

	Overall	Tanzania	Uganda	Zambia
**Participants (N = 59)**	**N = 59**	n = 23	n = 24	n = 12
Clinical officer, n (%)	14 (23.7)	5 (21.7)	7 (29.2)	2 (16.7)
Medical Officer, n (%)	8 (13.6)	3 (13.0)	5 (20.8)	0
Specialist Doctor, n (%)	1 (1.7)	1 (4.3)	0	0
Nurse, n (%)	36 (61.0)	14 (60.9)	12 (50)	10 (83.3)
**Facilitators (N = 17)**	**N = 17**	n = 8	n = 5	n = 4
Medical Officer, n (%)	5 (29.4)	3 (37.5)	1 (20.0)	1 (25)
Specialist Doctor, n (%)	5 (29.4)	2 (25)	2 (40.0)	1 (25)
Nurse, n (%)	7 (41.2)	3 (37.5)	2 (40.0)	2 (50)
**Participants' Facilities (N = 59)**	**N = 59**	n = 23	n = 24	n = 12
Health Centre, n (%)	17 (28.8)	9 (39.1)	6 (25)	2 (16.7)
District Hospital, n (%)	27 (45.8)	14 (60.9)	8 (33.3)	5 (41.7)
Regional Hospital, n (%)	4 (6.8)	0	4 (16.7)	0
Tertiary Referral Hospital, n (%)	9 (15.3)	0	4 (16.7)	5 (41.7)
Others,* n (%)	2 (3.4)	0	2 (8.3)	0
**Facilitators' Facilities (N = 17)**	**N = 17**	n = 8	n = 5	n = 4
Health Centre, n (%)	1 (5.9)	0	0	1 (25.0)
Regional Hospital, n (%)	1 (5.9)	0	0	1 (25.0)
Tertiary Referral Hospital, n (%)	14 (82.4)	8 (100)	5 (100)	1 (25.0)
Others,* n (%)	1 (5.9)	0	0	1 (25.0)

*Included faith based designated district hospitals

**Table 2:** Confidence ratings before and after course.

	Pre Course		Post Course		
Question	n (%)	CI (95%)	n (%)	CI (95%)	p value
Acutely ill adult	35 (42.7)	34.1–57.2	71 (86.6)	77.3–93.1	0.003
Acutely ill child	23 (28.0)	20.0–41.4	70 (85.4)	75.8–92.2	< .001
Injured adult	33 (40.2)	31.6–54.6	78 (95.1)	88.0–98.7	< .001
Injured child	25 (30.5)	22.2–44.1	68 (82.9)	73.0–90.3	< .001
Shock	39 (47.6)	39.0–62.2	77 (93.9)	86.3–98.0	< .001
Altered mental status	26 (31.7)	23.4–45.4	71 (86.6)	77.3–93.1	< .001
Difficulty in breathing	34 (41.5)	32.8–55.9	75 (91.5)	83.2–96.5	< .001
Emergency drugs	24 (29.3)	21.1–42.7	68 (82.9)	73.0–90.3	< .001
Obstructed airway management	26 (31.7)	23.4–45.4	73 (89.0)	80.2–94.9	< .001
Difficulty in breathing: Skills	32 (39.0)	30.4–53.4	77 (93.9)	86.3–98.0	< .001
Bleeding: Skills	36 (43.9)	35.3–58.5	77 (93.9)	86.3–98.0	< .001
Immobilization	19 (23.2)	15.6–35.8	24 (29.3)	19.7–40.4	0.451

Numbers indicate responses of ‘Very Confident’ in evaluating the given topic

**Fig 1:**
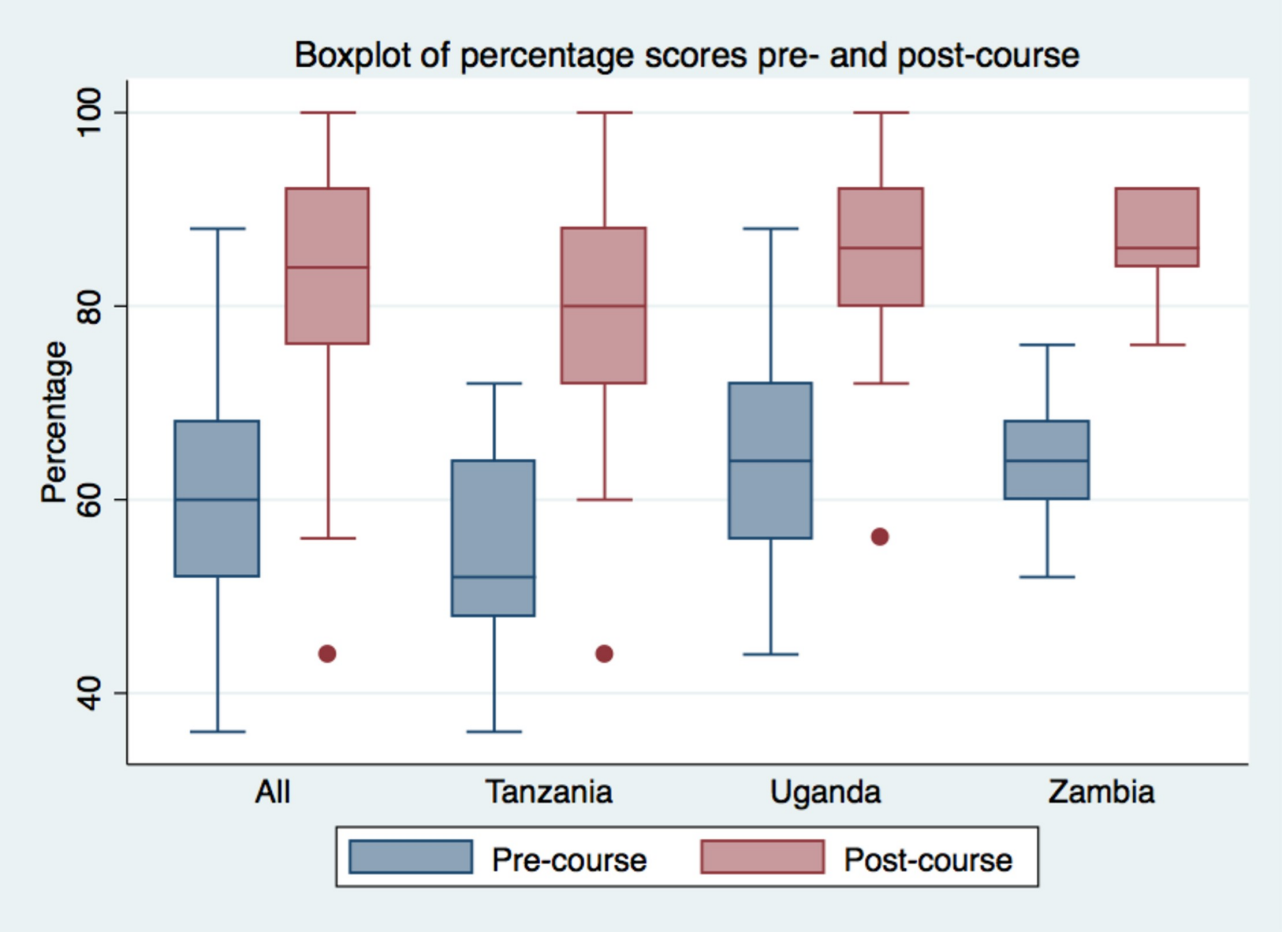
Pre- and post-course test results by country.

Pre-course exams from Ugandan and Zambian cohorts averaged approximately 64% correct and the Tanzanian cohorts averaged slightly lower at approximately 54%. Post-course exams were significantly improved, with the Tanzanian average increased to 79% (p<0.01), Ugandan average improved to 85% (p<0.01), and the Zambian average increased to 86% (p<0.01). (Table 2, Table 3 and Fig 1)

**Table 3:** Pre- and post-course test results.

	Pre-course score	Post-course score		
	Mean	Mean	Mean difference (95%CI)	p value
Tanzania (N = 23)	54	79	24 (20–29)	<0.001
Uganda (N = 24)	65	85	21 (16–26)	<0.001
Zambia (N = 12)	64	86	22 (17–27)	<0.001

Participants’ confidence levels in key emergency care areas were also surveyed. All areas (with the exception of immobilization) showed a statistically significant improvement in confidence levels. Confidence in immobilization skills did not reach significance, as many participants did not answer that question for unknown reasons.

### Survey results

Pre-course and post-course surveys from both participants and facilitators were evaluated. The pre-course survey included questions on desired learning goals as well as any concerns before the course. The post-course survey assessed course strengths, suggestions for improvement, whether the interviewee would recommend the course to others and which cadres of providers should receive the course, as well as any other comments. Table 4

**Table 4:** Perceived strengths and limitations of the BEC course with representative quotes.

Strengths	
Course content was useful	“I like all skills and knowledge to manage emergency cases/patient. I feel proud now after the course. I am going to help all patient accordingly” “Excellent training which puts emphasis on conditions that normally challenge health workers.” “The course covered almost all the areas about basic emergency skills and the science behind it.” “will really change people's mentality on how to handle clients” “For sure we will [save] many lives.”
Good course structure	“I like the teaching sessions, group discussions, and practical sessions. The time management was very good and course structure amazing.” “[I liked] the content of the course and the process of delivering the content.” “The course was very interactive; presentations well summarized; time well managed” “Clarity of language, interactive presentation by facilitators and skills (practical part of the course)” “best training [I] ever had”
Interactive sessions kept learners engaged	“[Interactive sessions] made the training not to be tiresome.”
Skills sessions were useful	“I liked the skills stations cause I was doing it imagining my hospital” “I have learnt a lot of skills in the management of emergency cases both in adult and paediatrics”
Course will save lives	“Thanks for this course teaching very educative and good life saving course” “This training is timely—very useful. It will help in reducing mortality and morbidity rates by giving timely interventions” “[T]his course was so useful to me and I am now confident of management emergency cases both adults and children.”
Much needed emergency care training	“We need more courses or assistance so as we can teach more people in our country or in many hospitals in our country.” “Emergency medicine is a fundamental skill in most of our limited resource settings that need[s] to be advocated for.” “I thank the organisers for this basic care course which is really important since we deal with different types of emergencies.”
Limitations	
Course delivery time is too short	“I think the time was kind limited, so maybe…let’s make 5 days for participants.”
No in-hospital practice	“If possible, I would recommend participants can go [to] casualty and demonstrate skills on real patients other than models only.”

#### Participant and facilitator expectations and concerns prior to the course

From the pre-course survey, the top three things participants and facilitators hoped to gain from the course were: improved knowledge of emergency care, improved clinical skills, and translation of this knowledge and skills into improved care for their patients. Facilitators also hoped to improve their teaching skills.

In terms of concerns, facilitators were most concerned about barriers to course dissemination beyond the initial training group, while participants were primarily concerned that the course might teach skills requiring equipment not available in their practice settings. One respondent expressed this as worry that facilitators would be “teaching us using equipment and materials we do not have in our district hospital setting.”

#### Perception on the strength and application of BEC in local context

After the course, the vast majority (75 of 76 facilitator and participant respondents) said that they would recommend the course to others.

**Course content was useful**: A salient theme on the strength of the course was that the content was very appropriate to local settings and would be useful to practitioners in their practice. One participant remarked that “the course covered almost all the areas about basic emergency skills and the science behind it.” Another wrote that the course “will really change people's mentality on how to handle clients,” and one respondent predicted that, “for sure we will [save] many lives.”

**Good course structure**: Facilitators and participants also felt that the organization and presentation style of the course were very clear and understandable and enjoyed the interactive nature of the course. “Clarity of language, interactive presentation by facilitators and skills (practical part of the course)” were identified as favourite parts of the course by one respondent. Other participants remarked, “I like the teaching sessions, group discussions, and practical sessions. The time management was very good and course structure amazing.” One described the course as the “best training [I have] ever had.”

**Interactive sessions kept learners engaged**: One respondent noted that the interactive sessions in the training were very useful to ensure continuous engagement. As noted by one participant, “[interactive sessions] made the training not to be tiresome.”

**Skills sessions were useful**: Most respondents listed specific clinical assessment and management skills when asked what they had learned in the course. “I have learnt a lot of skills in the management of emergency cases both in adult and paediatrics.” Other notable responses included general skills on how to stay calm in emergencies, the use of a systematic approach to patients, and that time is vital. Facilitators felt that they learned teaching skills, but also the importance of emergency care training.

**Course will save lives**: Both groups mentioned that the course demonstrated to them that emergency care can be performed in LMICs, and many commented that they feel the training will save lives. As one respondent noted, “This training is timely–very useful. It will help in reducing mortality and morbidity rates by giving timely interventions.” Many respondents also mentioned improved confidence: “this course was so useful to me, and I am now confident of management emergency cases both adults and children.”

#### Perception on the limitations of BEC

When asked what they would change about the course, nearly one third felt that the course should be longer in duration. One participant stated, “I think the time was kind [of] limited, so maybe…lets make 5 days for participants.” Eight participants also felt that the skills stations should be given more time, and three proposed that the course should involve practice on real patients. “If possible, I would recommend participants can go to casualty and demonstrate skills on real patients other than models only.” A little over one third of participants stated that they would not change anything. Other suggestions were to incorporate frequent refresher courses, to give the course for continuing medical education credit, and to incorporate the course into other training programmes, such as nursing and medical schools. The majority of respondents (73) felt that all healthcare providers would benefit from this course. Specific cadres named included nurses, doctors, clinical/medical officers, midwives, outpatient staff, paramedics/ambulance workers, and health attendants. One participant stated: “BEC should be incorporated in the teaching curriculum ranging from the nursing (diploma holder) to medical officer.” Some respondents also felt this course should be given to non-medical personnel, including police, army, taxi drivers, teachers and village health teams.

## Discussion

Overall, the course was very well received. Participants and facilitators highlighted the need for a course like the BEC that covers the essentials of assessing and managing emergency conditions. Participants felt that the course content and language level were appropriate. Both participants and facilitators reported a desire to hold further sessions of the course, and many requested that the trainings be disseminated widely throughout their countries. All of the pilot courses were successfully executed, and the course structure, demands, and logistics were deemed manageable by facilitators and participants.

Most participants felt the training was appropriate for providers in all lines of clinical work, and some mentioned that this training should be given to non-medical providers. This may represent the need for layperson first responder training, rather than the appropriateness of this particular course, which requires a basic knowledge of anatomy and physiology.

One of the stated goals of the BEC course was to deliver context-relevant education in the management of emergency conditions that is appropriate for limited-resource settings and not dependent on diagnostic or treatment interventions that may not be available. Indeed, one concern expressed by several respondents (participants and facilitators) prior to the course was that the instruction might presume resources that did not exist locally. This issue appears to have been successfully addressed by the course, as the most common sentiment regarding course content was that it was appropriate and useful.

Many respondents felt that the initial 4-day structure of the course was too rushed and felt that 5 days would be a preferred course length. These comments allowed the WHO course editors to adjust the recommended implementation plan, and in subsequent pilot and the final version of the BEC course, the schedule was adjusted to facilitate 5-day course delivery.

A notable result in our study was the statistically significant improvement in knowledge-based test performance. While this pilot study was not designed to evaluate long-term retention and change in clinical practice, it is clear that the course materials were sufficient to allow for significant knowledge acquisition. Facilitators also reported that the course materials were sufficient to prepare them to teach.

### Limitations

There were several limitations to this pilot study. First, all three sites tested were located in Eastern Sub-Saharan Africa. While the sites are representative of the region, the results may not be generalizable to countries in other areas of the world. Additionally, for the purposes of the pilot, an instructor/student ratio of approximately 1:4 was used, which allowed for more interaction between facilitators and participants and may have contributed to a better learning environment and improved knowledge acquisition. This ratio may not be feasible in all settings, and variable implementation may impact the reception and effectiveness of the course. Notwithstanding this, our results do characterize important findings about the acceptability and relevance of the course for its intended target audience.

After this pilot was completed, the BEC course was revised and distributed to experts in over 40 countries for additional peer review, and additional revisions were incorporated, so the findings from this pilot do not address the current version. These later changes, however, were generally limited to small content changes or additions, minor structural adjustments, or correction of linguistic errors. They did not affect the overall structure, essential content, language level, or logical approach taken by the course, which are the components primarily evaluated here.

Finally, and most importantly, this pilot does not address impact parameters such as long-term knowledge retention or translation to improved clinical practice. Further studies assessing the educational effectiveness and impact of the BEC will be critical to future dissemination and implementation.

## Conclusion

The BEC course content and delivery method were well-received by both participants and facilitators. This pilot demonstrates that a low-fidelity, openly available course taught by local instructors can be successful in knowledge transfer. We hope that these results will inform the efforts of implementers and developers of future courses. Further studies are needed to evaluate this course’s impact on clinical practice change and patient outcomes.

## Supporting information

S1 Table Table 1. BEC Course Programme.
(DOC)

S1 File Table 2. BEC Pre and Post Survey Questionnaire.
(DOCX)

## Abbreviations

ACLS: Advanced Cardiovascular Life Support
AFEM: African Federation for Emergency Medicine
BEC: Basic Emergency Care
BLS: Basic Life Support
LMIC: Low and Middle income countries
NCD: Non-Communicable Disease
WHO: World Health Organisation
