# Supplementary material for: Results from a World Health Organization pilot of the Basic Emergency Care Course in Sub Saharan Africa
Source: PLoS One. 2019 Nov 13;14(11):e0224257. doi: 10.1371/journal.pone.0224257 (PMC6853313; doi:10.1371/journal.pone.0224257)
Supplement: S1 Table — BEC Course Programme. (DOC) [file pone.0224257.s001.doc]

| **Country** | **Day 1** | **Day 2** | **Day 3** | **Day 4** |
| --- | --- | --- | --- | --- |
| Uganda  Tanzania | Introduction  Theoretical lectures: ABCDE, Trauma | Skills demonstration, practice, and assessment | Theoretical lectures: Shock, Difficulty in breathing | Theoretical lecture: Altered mental status  Final assessments |
| Zambia | Introduction  Theoretical lectures: ABCDE, Trauma | Theoretical lectures: Shock, Difficulty breathing | Skills demonstration, practice, and assessment | Theoretical lecture: Altered mental status  Final assessments |
