# Supplementary material for: Results from a World Health Organization pilot of the Basic Emergency Care Course in Sub Saharan Africa
Source: PLoS One. 2019 Nov 13;14(11):e0224257. doi: 10.1371/journal.pone.0224257 (PMC6853313; doi:10.1371/journal.pone.0224257)
Supplement: S1 File — BEC Pre and Post Survey Questionnaire. (DOCX) [file pone.0224257.s002.docx]

**BEC Survey-PRE**

1. Are you a Participant or Facilitator?

Participant Facilitator

2. What is your job title?

3. Where do you work?

Health Centre District Hospital Regional Hospital Tertiary Hospital

4. How long since you finished your first training?

5. How long in your current job?

6. How would you rate your confidence in the following?

A. Emergency management of the acutely ill adult

Very Confident Confident Fair Not confident at all Not sure

B. Emergency management of the acutely ill child

Very Confident Confident Fair Not confident at all Not sure

C. Emergency management of the injured adult

Very Confident Confident Fair Not confident at all Not sure

D. Emergency management of the injured child

Very Confident Confident Fair Not confident at all Not sure

E. Emergency management of the patient with Shock

Very Confident Confident Fair Not confident at all Not sure

F. Emergency management of the patient with altered mental status

Very Confident Confident Fair Not confident at all Not sure

G. Emergency management of the patient with difficulty in breathing

Very Confident Confident Fair Not confident at all Not sure

H. Understanding of emergency drugs

Very Confident Confident Fair Not confident at all Not sure

I. Have skills to manage an obstructed (blocked) airway

Very Confident Confident Fair Not confident at all Not sure

J. Have skills to manage a patient with difficulty breathing

Very Confident Confident Fair Not confident at all Not sure

K. Have skills to manage a patient with bleeding problems

Very Confident Confident Fair Not confident at all Not sure

L. Have the skills to immobilise patients

Very Confident Confident Fair Not confident at all Not sure

7. What do you expect to gain from this course?

8. Is there anything related to this course you are worried about?

9. Other comments?

**BEC Survey-POST**

1. Are you a Participant or Facilitator?

Participant Facilitator

2. What is your job title?

3. Where do you work?

Health Centre District Hospital Regional Hospital Tertiary Hospital

4. How long since you finished your first training?

5. How long in your current job?

6. How would you rate your confidence in the following?

A. Emergency management of the acutely ill adult

Very Confident Confident Fair Not confident at all Not sure

B. Emergency management of the acutely ill child

Very Confident Confident Fair Not confident at all Not sure

C. Emergency management of the injured adult

Very Confident Confident Fair Not confident at all Not sure

D. Emergency management of the injured child

Very Confident Confident Fair Not confident at all Not sure

E. Emergency management of the patient with Shock

Very Confident Confident Fair Not confident at all Not sure

F. Emergency management of the patient with altered mental status

Very Confident Confident Fair Not confident at all Not sure

G. Emergency management of the patient with difficulty in breathing

Very Confident Confident Fair Not confident at all Not sure

H. Understanding of emergency drugs

Very Confident Confident Fair Not confident at all Not sure

I. Have skills to manage an obstructed (blocked) airway

Very Confident Confident Fair Not confident at all Not sure

J. Have skills to manage a patient with difficulty breathing

Very Confident Confident Fair Not confident at all Not sure

K. Have skills to manage a patient with bleeding problems

Very Confident Confident Fair Not confident at all Not sure

L. Have the skills to immobilise patients

Very Confident Confident Fair Not confident at all Not sure

7. What did you like about the course?

8. What did you learn in the course?

9. What would you change about the course?

10. Would you recommend the course to others?

A. If yes- What type of provider?

11. Other comments?
